# Synapse Formation and Function Across Species: Ancient Roles for CCP, CUB, and TSP-1 Structural Domains

**DOI:** 10.3389/fnins.2022.866444

**Published:** 2022-04-25

**Authors:** Inés González-Calvo, Mélissa Cizeron, Jean-Louis Bessereau, Fekrije Selimi

**Affiliations:** ^1^Center for Interdisciplinary Research in Biology (CIRB), Collège de France, CNRS, INSERM, PSL Research University, Paris, France; ^2^Univ Lyon, Université Claude Bernard Lyon 1, CNRS UMR-5284, INSERM U-1314, MeLiS, Institut NeuroMyoGène, Lyon, France

**Keywords:** synapse, molecular conservation, CCP, CUB, TSP-1, invertebrates, vertebrates

## Abstract

The appearance of synapses was a crucial step in the creation of the variety of nervous systems that are found in the animal kingdom. With increased complexity of the organisms came a greater number of synaptic proteins. In this review we describe synaptic proteins that contain the structural domains CUB, CCP, or TSP-1. These domains are found in invertebrates and vertebrates, and CUB and CCP domains were initially described in proteins belonging to the complement system of innate immunity. Interestingly, they are found in synapses of the nematode *C. elegans*, which does not have a complement system, suggesting an ancient function. Comparison of the roles of CUB-, CCP-, and TSP-1 containing synaptic proteins in various species shows that in more complex nervous systems, these structural domains are combined with other domains and that there is partial conservation of their function. These three domains are thus basic building blocks of the synaptic architecture. Further studies of structural domains characteristic of synaptic proteins in invertebrates such as *C. elegans* and comparison of their role in mammals will help identify other conserved synaptic molecular building blocks. Furthermore, this type of functional comparison across species will also identify structural domains added during evolution in correlation with increased complexity, shedding light on mechanisms underlying cognition and brain diseases.

## Introduction

The emergence of synapses is an ancient process which is not fully elucidated (Arendt, 2020). Much of our knowledge comes from comparing synaptic proteins in different animal species. The ancestor of chemical synapses, the Ursynapse, appeared in the common ancestor of cnidarians and bilaterians and some synaptic proteins, named protosynaptic proteins, existed before the emergence of the Ursynapse (Ryan and Grant, 2009). Since many synaptic proteins are shared by all bilaterians, studies carried in invertebrate animal models have proven very useful to identify and characterize key synaptic proteins. For instance, historical genetic screens conducted in *Caenorhabiditis elegans* by Sydney Brenner identified many genes required for synaptic function, such as *unc-13*, which encodes a protein essential for synaptic vesicle exocytosis (Brenner, 1974). The early identification of *unc-13* enabled the identification of several vertebrate orthologues with functional conservation and involvement in genetic diseases. Conversely, studies in vertebrates, in particular using proteomics, have revealed a considerable diversity of synaptic proteins (Bayés et al., 2011). This diversity arises at least in part from the two rounds of whole-genome duplication events that occurred at the base of the chordate lineage (Van de Peer et al., 2009). Some paralogs, such as members of the DLG (disk large homolog) family that are localized at the postsynaptic density, acquired specific functions (Nithianantharajah et al., 2013) and specific spatiotemporal expression patterns (Zhu et al., 2018; Cizeron et al., 2020). Other synaptic proteins, such as the GABAergic postsynaptic scaffolds Gephyrin and Collybistin, are present in vertebrates but are absent in *Caenorhabiditis elegans* (Arendt, 2020). Comparing the synaptic machineries across species is thus a powerful approach to understand the molecular basis of synaptic complexity in vertebrates.

**FIGURE 1:**
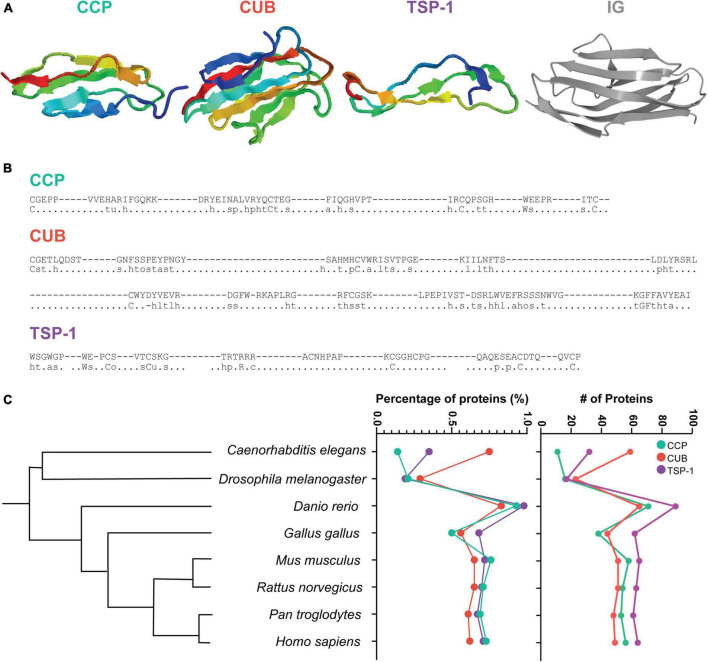
CCP, CUB, and TSP-1 domains. **(A)** Three-dimensional structure of a CCP domain (from rat GABA B Receptor 1a (Blein et al., 2004), PBD id:1srz), a CUB domain (from human tumor necrosis factor-inducible gene 6 protein (Briggs et al., 2015), PDB id: 2wno), a TSP-1 domain (from rat F-spondin (Pääkkönen et al., 2006), PDB id: 1szl) and a IG domain (from mouse NCAM (Jensen et al., 1999), PDB id: 3ncm). Adapted from ebi.ac.uk/pdbsum **(B)** For each domain, an example is given (top sequence: β-2-glycoprotein 1 from Bos Taurus (PGCA_BOVIN/1-5) for the CCP domain; bone morphogenetic protein 1 from Homo sapiens (BMP1_HUMAN/1-1) for the CUB domain and Properdin from Homo sapiens (PROP_HUMAN/2-0) for the TSP-1 domain), as well as the consensus terms (80%, bottom sequence). Adapted from smart.embl.de. **(C)** Number and percentage of proteins containing CCP, CUB, or TSP-1 domains (green, red and purple, respectively, right) for different species ordered via a phylogenetic tree (left). Data from SMART genomic mode domain evolution (smart.embl.de) and the interactive Tree of Life (itol.embl.de).

Certain synaptic proteins were initially characterized for their function outside the central nervous system (CNS). The seminal work of the laboratory of Dr. Carla Shatz, followed by others, showed that proteins of the immune system are also expressed by neurons, are regulated by activity and contribute to synapse development and function (for a review cf. (Boulanger, 2009)). One particular category of immune-related proteins with neuronal function are the proteins of the complement system implicated in innate immunity or their regulators: the complement proteins C1q and C3 contribute to synaptic pruning in the developing brain (Stephan et al., 2012) and the complement inhibitor SUSD4 (Sushi domain-containing protein 4) regulates synaptic plasticity (González-Calvo et al., 2021). Invertebrates do not have a complement system or an adaptative immune system. Yet they express proteins with domains typically found in proteins of these systems, such as the immunoglobulin (Ig) domain or the Complement Control Protein domain (Figure 1). During evolution these structural domains may have been used for establishing neuronal synapses before being used to build the complement or adaptative immune systems. Thus, rather than thinking about entire proteins, identifying the presence of specific structural domains and the function of the corresponding proteins at neuronal synapses across species might provide an insight into the mechanisms that control core aspects of the development and function of neuronal synapses. In particular, it might help understand how the high diversity of synapse types needed to produce large neuronal networks in vertebrates was attained.

In line with this idea, Vogel and Clothia studied 38 eukaryotic genomes, ranging from unicellular organisms to mammals, and tested the correlation between the number of proteins in superfamilies characterized by specific structural domains and the complexity of the organisms as defined by the number of cell types (Vogel and Chothia, 2006). They identified 194 superfamilies with a correlation ≥ 0.80 (Vogel and Chothia, 2006): these included the Ig domain containing superfamily (correlation: 0.97), but also several other superfamilies of proteins with immune-related roles such as the CCP (Complement Control Protein, also known as Sushi or the short consensus repeats (SCR) domain), CUB (complement C1r/C1s, Uegf, Bmp1), and TSP-1 (thrombospondin type-1) domains (correlation 0.94, 0.84, 0.91, respectively). Like the Ig domain, these three domains possess a structure characterized by sandwich like folds that might favor protein-protein and protein-glycan interactions (Zinn and Özkan, 2017) (Figure 1). The CCP, CUB, and TSP-1 domains have been previously identified in synaptic proteins in C. elegans prompting us to review the role of these three superfamilies in different species. The role of the Ig superfamily in the nervous system has been addressed in several reviews recently (Sytnyk et al., 2017; Zinn and Özkan, 2017; Cameron and McAllister, 2018; Sanes and Zipursky, 2020).

## Key Synaptic Roles for the TSP-1, CCP, and CUB Structural Domains Identified in Invertebrates

Genetic screens in *Caenorhabditis elegans* were instrumental for the identification of new synaptic proteins and their function at the neuromuscular junction (NMJ). In particular, screens using the anthelmintic drug levamisole identified two genes, *lev-9* and *lev-10*, coding for synaptic proteins required for the aggregation of levamisole-sensitive cholinergic receptors (L-AChRs) on muscle cells (Gendrel et al., 2009). The LEV-9 and LEV-10 proteins are characterized by a specific structural domain: CCP (Complement Control Protein, also known as Sushi or the short consensus repeats (SCR) domain) and CUB (complement C1r/C1s, Uegf, Bmp1) domains (Figure 1), respectively. Later, *madd-4* was identified as a gene encoding a protein composed of up to 10 thrombospondin type-1 (TSP-1) domains (Figure 1), and necessary for the correct localization of LEV-9/LEV-10/L-AChR complexes. Thus, CCP, CUB, and TSP-1 domains have important roles at *C. elegans* NMJs.

The TSP-1 domain (approximately 60 amino acids in length, also known as TSR-1 repeat domain or TSR) was first described in thrombospondins, multimeric glycoproteins present at the cell surface and in the extracellular matrix. It is composed of an antiparallel, three-stranded fold with a positively charged groove (Tan et al., 2002). The CCP domain was first described in the human complement component factor B. It is also found in several complement-related proteins like the complement receptor C1R, the complement protein C2 and the serine proteases MASP1-3 to list a few (cf. Annex 1 for the list of CCP containing proteins expressed in the human brain). The CCP consensus sequence spans ∼60 residues and contains hydrophobic residues forming a β-sheet core held together *via* disulphide bridges between four cysteine residues that are conserved in 80% of the sequences (Figure 1; Reid and Day, 1989). The CUB domain was first identified in the complement subcomponent C1r/C1s, sea urchin protein Uegf, and BMP-1 proteins. It spans about 100-110 residues (Bork and Beckmann, 1993). The consensus sequence (Figure 1) often comprises four conserved cysteines that can form two disulfide bounds, as well as conserved hydrophobic and aromatic positions that organize into a compact ellipsoidal ß-sandwich (Bork and Beckmann, 1993; Varela et al., 1997).

All three domains were present in the last common ancestor of eumetazoans, as shown by their presence in bilaterians (described in this review) and in cnidarians (SMART website). Proteins containing these structural domains can thus be expected to play ancient and key roles in the building and function of neuronal circuits.

### The TSP-1 Domain Containing Protein MADD-4 and Synapse Type Specification

**FIGURE 2:**
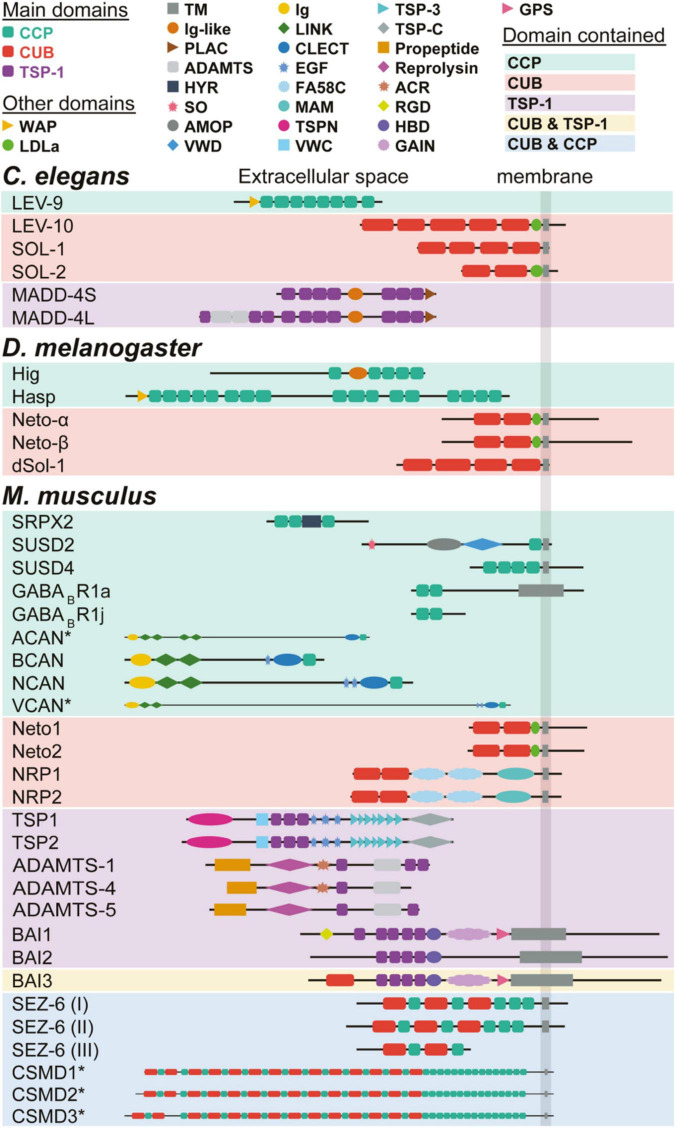
CCP, CUB, and TSP-1 domain-containing proteins with known synaptic functions in *C. elegans*, *D. melanogaster* and *M. musculus*. Domain structure is indicated for each protein and proteins are grouped depending on their CCP, CUB, and/or TSP-1 domain content. * indicates a 1:2 scale for the protein.

In *C. elegans*, the NMJ has been used as a model to study synapse specificity on body-wall muscle cells, which receive both excitatory (ACh) and inhibitory (GABAergic) inputs. MADD-4, a TSP-1 domain containing protein, is secreted presynaptically by motoneurons and defines the identity of postsynaptic domains by controlling the clustering of cholinergic receptors (AChRs) and GABAergic A receptors (GABA_A_Rs) in front of the corresponding neurotransmitter release sites (Pinan-Lucarré et al., 2014; Zhou et al., 2020). The *madd-4* locus generates three MADD-4 isoforms through the use of alternative promoters and alternative splicing. The two long MADD-4L isoforms (MADD-4A and MADD-4C) differ by 2 amino acids and are composed of 10 TSP-1 repeats, an ADAMTS (a disintegrin and metalloproteinase with thrombospondin motifs) cysteine-rich module, an ADAMTS spacer module, an Ig-like C2-type domain and a PLAC (Protease and LACunin) domain (Figure 2). The MADD-4S isoform is identical to the C-terminal moiety of MADD-4L and contains only 7 TSP-1 repeats, an Ig-like C2-type domain and a PLAC domain (Figure 2). MADD-4L is exclusively expressed by cholinergic motoneurons and triggers the clustering of AChRs. MADD-4S is expressed by both cholinergic and GABAergic motoneurons. At GABAergic NMJs, it promotes the recruitment of GABA_A_Rs. At cholinergic NMJs, it prevents the inappropriate recruitment of GABA_A_Rs. In the absence of MADD-4 protein, both AChR and GABA_A_R clusters relocalize to extrasynaptic areas (Pinan-Lucarré et al., 2014). Hence, the repertoire of isoforms expressed by specific MNs controls post-synaptic identity (Pinan-Lucarré et al., 2014). Recently, a study of *madd-4* null mutants showed that MADD-4 also controls the timing of synapse remodeling during development (Chen et al., 2021).

The control of synapse specification by MADD4 isoforms requires the control of distinct molecular pathways for each type of receptors. MADD-4L allows the correct positioning of LEV-9/LEV-10/L-AChRs complexes (cf. below) in front of cholinergic boutons (Pinan-Lucarré et al., 2014). MADD-4S positions GABARs by two parallel pathways. First, it localizes Neuroligin-1 (NLG-1) at GABA postsynaptic sites through direct interactions (Maro et al., 2015; Tu et al., 2015). Second, it activates the netrin receptor UNC-40/DCC (Tu et al., 2015), which triggers the formation of an intracellular scaffold involving LIN-2/CASK and FRM-3/FARP (Zhou et al., 2020). This intracellular scaffold promotes the recruitment of GABA_A_Rs onto NLG-1 clusters. In addition, the mechanism allowing the dual function of MADD-4S at cholinergic versus GABAergic synapses remains to be understood, but probably involves dimerization with the long isoform MADD-4L. Finally, both MADD-4 isoforms promote the synaptic localization of the heparan sulfate glycoprotein syndecan/SDN-1, a key-component of cholinergic NMJs (Zhou et al., 2021). The specific role of the TSP-1 domains in these various molecular interactions remains to be determined.

### CCP Containing Proteins and Neurotransmitter Receptor Clustering

#### LEV-9 (*Caenorhabiditis elegans*)

LEV-9 is a secreted protein composed of eight CCP domains and one WAP (whey acidic protein) domain (Figure 2). L-AChRs are undetectable at NMJs of *lev-9* mutants, while ACh presynaptic boutons and postsynaptic GABA_A_Rs are normally distributed (Gendrel et al., 2009). Surprisingly, biochemical and electrophysiological experiments demonstrated that L-AChRs are expressed at normal levels in *lev-9* mutants, highlighting that it is dispensable for correct expression and surface targeting of functional L-AChRs (Gendrel et al., 2009). However, the evoked response after stimulation of AChRs was reduced and presented an increased time to peak and decay time in *lev-9* mutants (Gendrel et al., 2009). These data supported the presence of declustered L-AChRs in the absence of LEV-9. LEV-9 is expressed by muscles, where it functions cell-autonomously, and localizes to cholinergic NMJs (Gendrel et al., 2009). LEV-9 activation by C-terminal cleavage is required for proper L-AChR clustering but not for LEV-9 secretion (Briseño-Roa and Bessereau, 2014). The WAP domain from LEV-9 is dispensable for its role in L-AChR clustering (Gendrel et al., 2009), indicating that the CCP domains are the main actors of LEV-9 clustering function.

#### Hig and Hasp (*Drosophila melanogaster*)

Two CCP domain-containing proteins with similar function in invertebrates have been studied in *Drosophila melanogaster*: Hikaru genki (Hig) and Hasp (Hig-anchoring scaffold protein). The Hig protein contains five CCP domains and one immunoglobulin (Ig)-like domain, while Hasp contains one WAP domain followed by up to seventeen CCP domains (Figure 2). Like LEV-9, Hig and Hasp are processed, but the identity of the functionally important fragment is unknown (Nakayama et al., 2016). Both *hig* and *hasp* mutants have reduced locomotion and longevity (Hoshino et al., 1993; Nakayama et al., 2016). In the adult brain, Hig and Hasp mostly localize to the synaptic cleft of cholinergic synapses, where they occupy distinct, overlapping areas (Nakayama et al., 2014, 2016). When expressed ectopically, Hig is able to diffuse in the extracellular space and is trapped at cholinergic synaptic clefts (Nakayama et al., 2014, 2016). Analysis of loss of function mutants (Nakayama et al., 2014, 2016) shows that Hasp interacts with Hig to anchor it at the synaptic cleft of cholinergic synapses, and that the localization of Hig at synapses is reciprocally dependent on AChRs (Nakayama et al., 2016). Thus, Hig is functionally similar to LEV-9 and controls AChR clustering at synapses in *D. melanogaster*.

### CUB Containing Proteins and Regulation of Neurotransmitter Receptor Clustering and Properties

#### LEV-10 (*Caenorhabiditis elegans*)

The first discovery of the involvement of the CUB domain in the regulation of ionotropic receptors came from the identification of *lev-10* (Gally et al., 2004). LEV-10 is a type 1 transmembrane protein composed of an extracellular LDLa (low-density lipoprotein receptor domain class A) domain and five extracellular CUB domains (Figure 2). *Lev-10* mutants present similar phenotypes to *lev-9* mutants: *lev-10* is dispensable for expression and membrane targeting of L-AChRs, but its absence leads to declustering and redistribution of the receptors to extrasynaptic areas (Gally et al., 2004). Like LEV-9, LEV-10 is expressed in muscles, distributes to cholinergic NMJs and functions cell-autonomously (Gally et al., 2004). In the absence of LEV-9, LEV-10 or L-AChRs, the other proteins fail to localize to cholinergic NMJs, showing that they form a tripartite complex necessary for their mutual clustering (Gally et al., 2004; Gendrel et al., 2009). An additional partner contributes to this extracellular scaffold: OIG-4 contains a single immunoglobulin domain and stabilizes the interaction between L-AChRs and LEV-10 (Rapti et al., 2011). The extracellular part of LEV-10 is sufficient to rescue L-AChR clustering in *lev-10* mutants and the LDLa domain is dispensable (Gally et al., 2004; Rapti et al., 2011), highlighting the role of LEV-10 CUB domains in L-AChR clustering.

#### SOL-1 and SOL-2 (*Caenorhabiditis elegans*)

In *C. elegans*, two other CUB domain-containing proteins have known synaptic functions: SOL-1 and SOL-2. Both proteins regulate the channel properties of the glutamate receptor GLR-1, an ortholog of AMPA receptors (Walker et al., 2006; Zheng et al., 2006, 2004; Wang et al., 2012). SOL-1 and SOL-2 are both type 1 transmembrane proteins that differ by their extracellular domain: SOL-1 contains four extracellular CUB domains and SOL-2 one LDLa and two CUB domains (Figure 2). These proteins were both discovered in genetic screens looking for suppressors of the *lurcher* phenotype, a hyperreversal behavior caused by hyperactive AMPA receptors in a specific *glr-1* mutant. *sol-1* and *sol-2* mutants, like *glr-1* mutants, present defects in tactile avoidance, duration of forward movement and show delayed response to a drop of hyperosmotic solution (Zheng et al., 2004; Wang et al., 2012). These behavioral phenotypes are accompanied by defects in synaptic transmission: glutamate and kainate-evoked currents are reduced while NMDA-evoked currents are unchanged (Zheng et al., 2004; Wang et al., 2012). SOL-1 and SOL-2 function cell autonomously in AVA neurons (Zheng et al., 2004; Wang et al., 2012). The third CUB domain is critical for SOL-1 function as shown by rescue experiments using different mutant constructs (Zheng et al., 2006). Synaptic defects are not due to changes in expression levels or surface expression of GLR-1 complexes (Zheng et al., 2004; Walker et al., 2006; Wang et al., 2012). Rather, SOL-1 increases glutamate currents by slowing GLR-1 desensitization kinetics and by increasing its recovery from desensitization (Walker et al., 2006). This role in the control of receptor properties is further supported by experiments in *Xenopus* oocytes showing that the minimal composition for a functional GLR-1 complex is constituted by the GLR-1 subunit, one TARP (Transmembrane AMPA receptor Regulatory Protein) homolog (STG-1 or STG-2) and SOL-1 (Walker et al., 2006; Wang et al., 2012). Indeed, GLR-1, SOL-1 and SOL-2 colocalize in puncta that distribute along the processes of AVA neurons (Wang et al., 2012). While a soluble version of SOL-1 (s-SOL-1), containing only its extracellular part (i.e., 4 CUB domains), is sufficient to partially rescue the behavioral and physiological defects in *sol-1* mutants (Zheng et al., 2006), it requires the presence of SOL-2 to function, indicating that SOL-2 may be involved in linking SOL-1 to the GLR-1 complex (Wang et al., 2012). Rescue experiments in *sol-2* mutants showed an additional SOL-1-independent role of SOL-2 in modulating GLR-1 gating (Wang et al., 2012). Thus, SOL-1 and SOL-2 are two auxiliary subunits of GLR-1 that differentially regulate its gating and SOL-2 has an additional structural role in bridging the GLR-1 receptor to SOL-1.

#### Neto (*Drosophila melanogaster*)

The *D. melanogaster* Neto has a domain composition similar to *C. elegans* SOL-2: it contains a type I transmembrane domain, two extracellular CUB domains and a LDLa motif (Figure 2). Alternative splicing generates two different isoforms, Neto-ɑ and -β, which differ by their intracellular domains (Han et al., 2015; Ramos et al., 2015). In *D. melanogaster*, *neto* is an essential gene, as null mutations result in paralyzed embryos that never hatch into larval stages, while hypomorphic alleles cause severe locomotor defects (Kim et al., 2012). Although Neto-β is the predominant isoform at glutamatergic NMJs (Ramos et al., 2015; Han et al., 2020), expression of either Neto isoform in muscles rescues locomotion and allows the development of viable and fertile adults (Kim et al., 2012; Ramos et al., 2015). The importance of the extracellular CUB and LDLa domains is highlighted by the fact that muscle expression of a chimera, in which the intracellular part is replaced by GFP, can rescue lethality and paralysis in *neto* mutants (Ramos et al., 2015). Neto mostly localizes at type I (glutamatergic) neuromuscular junctions, where it colocalizes with ionotropic glutamate receptors (iGluRs) at postsynaptic densities (Kim et al., 2012). Neto is dispensable for normal levels and surface expression of iGluRs (Kim et al., 2012, 2015). In the absence of Neto, synapses form normally at the prepatterning stage but iGluRs fail to cluster at postsynaptic densities and maintenance of the postsynaptic site is altered (Kim et al., 2012). This is accompanied by electrophysiological defects in miniature and evoked excitatory junctional potentials (Kim et al., 2012). In contrast, when *neto* is over-expressed, it accumulates at extrajunctional sites and results in synapse defects, probably due to extrajunctional trapping of iGluRs (Kim et al., 2015). In iGluR mutants, Neto clusters do not form, indicating a codependency of iGluRs and Neto (Kim et al., 2012), which is reminiscent of the dependent clustering of LEV-10 with L-AChRs in *C.elegans* and Hig/Hasp with AChRs in *D. melanogaster*. Co-expression of Neto-ɑ or Neto-β with iGluR subunits in *Xenopus* oocytes greatly enhances receptor currents, suggesting that the clustering mechanism may enhance receptor function (Han et al., 2015). The two isoforms have distinct roles. Neto-β, *via* its intracellular domain, regulates the composition of the postsynaptic side at NMJs, by promoting a preferential recruitment of GLURIIA over GLURIIB (Ramos et al., 2015). Neto-β is also involved in regulating the postsynaptic structure by promoting the formation of subsynaptic reticulum (Ramos et al., 2015). On the postsynaptic side, Neto-ɑ limits the size of the receptor fields. On the presynaptic side, the extracellular domains of Neto-ɑ are sufficient for the modulation of basal neurotransmission and its intracellular domain regulates presynaptic homeostasis (Han et al., 2020). Thus, the common extracellular domains, including the CUB domains, of the two Neto isoforms modulate channel properties while the divergent intracellular domains control specific functions of Neto proteins in regulating synapse composition and homeostasis.

#### dSol-1 (*Drosophila melanogaster*)

dSol-1 is another CUB domain-containing protein recently identified in a screen looking for genes regulating presynaptic homeostasis at the drosophila NMJs (Kiragasi et al., 2020). It has a structure very close to *C. elegans* SOL-1 with four extracellular CUB domains and a transmembrane domain (Figure 2). Interestingly, both dSol-1 or *C. elegans* SOL-1 are able to enhance GLR-1 receptor function in heterologous cells (Walker et al., 2006), highlighting the functional conservation of the two proteins. *dSol-1* is expressed in the nervous system and excluded from postsynaptic muscles (Kiragasi et al., 2020). At drosophila NMJs, proper baseline transmission and presynaptic homeostatic potentiation requires the presynaptic Kainate-type ionotropic glutamate receptor subunit 1D KaiR1D (Kiragasi et al., 2020). Loss of function and rescue experiments showed the presynaptic requirement of dSol-1 for these two aspects (Kiragasi et al., 2020), and in addition, suggest that *dSol-1* may directly modulate the function of KaiR1D to enhance basal neurotransmitter release (Kiragasi et al., 2020).

## Conservation and Extension of Ancient Functions in Central Synapses of Mammals

The percentage and absolute numbers of proteins containing CCP and TSP-1 domains increases in vertebrates compared to invertebrates (Figure 1 and smart.embl.de). For example, the number of proteins containing at least one CCP domain in *C. elegans* is 11, representing 0.14% of the total pool of proteins, while in *Rattus norvegicus*, 54 CCP-containing proteins are found, representing 0.71% of the total pool of proteins. This suggests a correlation between the number of proteins containing these domains and organism complexity. This relationship is true for the CUB domain when numbers are compared with *D. melanogaster*, but not with *C. elegans* where a large number of CUB domain-containing proteins are found, suggesting a specific evolution in this organism (Figure 1)^1^. Many of these proteins have yet to be studied functionally and only a few have been identified to play roles at neuronal synapses in mammals.

### TSP-1 Domain-Containing Proteins and Synaptogenesis

#### Thrombospondins

The Thrombospondin family is composed of five different members (TSP1-5), which are large secreted extracellular matrix (ECM) proteins that mediate cell–cell and cell–ECM interactions (reviewed in (Ferrer-Ferrer and Dityatev, 2018)). Amongst them, TSP1 and TSP2 are characterized by the presence of three TSP-1 domains, in addition to the Type 2 and Type 3 repeats common to all thrombospondins (Adams and Tucker, 2000; Figure 2). They are secreted by astrocytes and promote synapse formation in various neuronal types and networks (Christopherson et al., 2005). All TSPs have a synaptogenic effect in cultured retinal ganglion cells, indicating that this function is independent of the TSP-1 domain (Eroglu et al., 2009). *Tsp1* and *Tsp2* double knockout mice present a reduction of 50% of excitatory synapse numbers in the cortex at postnatal day P8 and a reduction of around 30% is still found by P21 (Christopherson et al., 2005). Both TSP1 and TSP2 can increase synapse numbers in cultured retinal ganglion cells. While those TSP-induced synapses are ultrastructurally normal and presynaptically active, they are postsynaptically silent (Christopherson et al., 2005). In the mouse inner ear, TSP1 and TSP2 are also required for afferent synaptogenesis and synapse function and they are only partially functionally redundant (Mendus et al., 2014).

TSPs have many interacting partners (reviewed in (Risher and Eroglu, 2012)), including synaptic proteins. TSP1 can bind to beta1-integrins *via* its TSP-1 domains (Calzada et al., 2004). Beta-integrins control the accumulation of GlyRs at inhibitory synapses in cultured spinal cord neurons and TSP1 can reduce their mobility and increase their accumulation at synapses as well (Charrier et al., 2010). TSP1 also reduces the accumulation of AMPA receptors at synapses in spinal cord neurons (Hennekinne et al., 2013). Furthermore, TSP1 can inhibit the accumulation of AMPA-type glutamate receptors at synapses induced by PTX-3 (Fossati et al., 2019), independently of the synaptogenic domain identified by Eroglu et al. These results suggest a dual function of TSP1, on one hand in promoting synapse formation, independent of its TSP-1 domains, and on the other hand in putting a brake on receptor accumulation at synapses *via* its TSP-1 domain.

#### The Adhesion Receptors BAI1, BAI2, and BAI3

The three brain-specific angiogenesis inhibitor receptors (BAI1-3, Figure 2) are a subgroup of the adhesion-GPCR family of receptors (Sigoillot et al., 2016; Scholz et al., 2019). They all share the same organization: an intracellular domain with a PDZ binding domain, a seven transmembrane domain, a proteolytic cleavage site, a hormone binding domain and a long extracellular domain with four TSP-1 domains for BAI2 and BAI3 and five for BAI1. Particularities are the presence of a CUB domain at the N-terminus of BAI3 and an RGD domain at the N-terminus of BAI1, indicating that these receptors might not be completely functionally redundant.

BAI1 is enriched in biochemical preparations of postsynaptic densities (Duman et al., 2013; Stephenson et al., 2013), and besides interacting with proteins regulating the cytoskeleton, can interact with several synaptic proteins such as PSD95 intracellularly (Stephenson et al., 2013) and Neuroligin-1 extracellularly (Tu et al., 2018). Knockdown of BAI1 in hippocampal neurons leads to reduced spine density and immature spine morphogenesis both in primary cultures and *in vivo* (Duman et al., 2013; Tu et al., 2018). BAI1 can induce the clustering of the VGluT1 presynaptic vesicle protein in a co-culture assay through its extracellular domain, and both this function and its synaptogenic ability in cultured hippocampal neurons necessitate the N-terminal TSP-1 containing domain (Tu et al., 2018). No deficits in spinogenesis were found in hippocampal neurons of BAI1 knockout mice (Zhu et al., 2015), suggesting a potential non-cell autonomous compensation. However, knockout of BAI1 in mice leads to a decrease in PSD95 protein levels and PSD thickness and a deficient long-term synaptic plasticity in CA1 hippocampal neurons (Zhu et al., 2015).

The first role described for the BAI3 receptor in neurons was the regulation of dendritogenesis in cerebellar Purkinje cells (Lanoue et al., 2013). BAI3 was further shown to promote spinogenesis and synaptogenesis in cerebellar Purkinje cells (Sigoillot et al., 2015), and in the olfactory bulb (Wang et al., 2020). BAI3 binds to the globular domain (gC1q) of the C1QL subfamily of C1q-related proteins *via* both the TSP-1 domains and the CUB domain (Bolliger et al., 2011; Kakegawa et al., 2015). Loss-of-function studies have shown that C1QL proteins are required for proper synaptogenesis in the cerebellum (Kakegawa et al., 2015; Sigoillot et al., 2015), amygdala and cortex (Martinelli et al., 2016) and olfactory bulb (Wang et al., 2020). The CUB domain of BAI3 is indeed essential for BAI3 synaptic function in cerebellar Purkinje cells, since a mutant lacking this domain cannot rescue the BAI3 loss-of-function phenotype (Kakegawa et al., 2015). Since C1QL proteins are secreted, it is probable that C1QL proteins form a bridge between BAI3 receptors postsynaptically and a membrane protein presynaptically, in a manner similar to the neurexin-CBLN-GluD2 tripartite complex. Indeed, C1QL2 and C1QL3 can bind to neurexin 3 (Matsuda et al., 2016) and a very recent study suggests the formation of a tripartite complex between neuronal pentraxin-1, C1QL3 and BAI3 (Sticco et al., 2021). Very recently, BAI1 and BAI3 receptors were shown to promote synapse formation via transsynaptic binding of RTN4 receptors (Wang et al., 2021). One of the TSP-1 domains in BAI receptors, and its glycosylation, is essential for this binding (Wang et al., 2021), highlighting the importance of TSP-1 domains for synapse formation.

### CCP Domain: From Receptor Trafficking to Synaptic Plasticity

#### GABA_B_ Receptors

Metabotropic GABA_B_ receptors mediate slow inhibitory transmission in the central nervous system. These receptors are heterodimers composed of GABA_B_R1 and GABA_B_R2 subunits. There are two major R1 splice variants, R1a and R1b, that differ by the presence of two CCP domains in the N terminus of R1a (Figure 2). Several other R1 splice variants contain CCP domains but no transmembrane domain, indicating that they are secreted (Lee et al., 2010). The CCP domains of R1a are sufficient to target an otherwise diffuse protein to axons, suggesting that the CCP domains of R1a function as an axon targeting signal (Biermann et al., 2010). Furthermore, these CCP domains confer greater surface stabilization to R1a/R2 GABA_B_ receptors compared to R1b/R2 and are sufficient when fused to mGluR2 receptors to increase their surface stability (Hannan et al., 2012). The isoforms R1a and R1b also differ in their probability to diffuse laterally at postsynaptic sites: R1a/R2 are more mobile than R1b/R2 (Hannan et al., 2016). The R1j isoform encodes the signal peptide and the two CCP domains of R1a (Figure 2). A recombinant protein mimicking this isoform can bind to neuronal membranes and impairs the inhibitory effect of GABA_B_ receptors on glutamate release but does not affect the activity of the GABA_B_ receptors (Tiao et al., 2008). CCP domains can thus regulate the axonal trafficking and surface stabilization of GABA_B_ receptors as well as their function at excitatory synapses. These functions involves the interaction of the first CCP domain of GABA_B_R1a with APP, AJAP-1 and PIANP (Schwenk et al., 2016; Dinamarca et al., 2019), further highlighting the functional importance of CCP domains in the regulation of synaptic protein localization and synapse function.

#### SRPX2

SRPX2 (Sushi-repeat protein X-linked 2) was identified as the causal gene for Rolandic (also known as Sylvian) epilepsy (Roll et al., 2006) and encodes a 465 amino acid secreted protein containing three CCP domains and one hyaline repeat (Figure 2). The hyaline repeat domain folds similarly to the IG-like domain found in the protein Hig from *Drosophila*. The Y72S mutation identified in patients with Rolandic epilepsy is in the immediate vicinity of a cysteine residue that is predicted to participate in the disulfide bond of the SRPX2 CCP domain (Roll et al., 2006), showing the importance of CCP domains for the brain function of SRPX2.

In rodents, *Srpx2* is expressed during the development of the cortex (Salmi et al., 2013). In utero *Srpx2* gene silencing in rats leads to impaired neuronal migration, altered positioning of projection neurons and altered dendritogenesis, together with a dramatic increase in glutamatergic and GABAergic spontaneous burst-type activities (Salmi et al., 2013), in line with seizures in humans with *SRPX2* mutations. However, other studies in the mouse, using knockdown or knockout models, and dissociated cultured neurons, support a role for SRPX2 in promoting excitatory synapse formation and function in various regions of the cortex (Sia et al., 2013; Cong et al., 2020). Indeed, mice lacking SRPX2 expression have decreased spine density and synapse numbers in the retinogeniculate pathway and in the somatosensory cortex and a decreased maximum AMPA receptor current at retinogeniculate synapses (Cong et al., 2020). Cong et al. (2020) also showed that SRPX2 can interact with the complement protein C1Q, and that it inhibits complement C3 accumulation and microglial-mediated synapse elimination (Cong et al., 2020). These data show that SRPX2 promotes excitatory synapse formation at least in part by inhibiting synapse elimination. Thus, the exact role of SRPX2 and the mechanism leading to epilepsies remains to be determined. A yeast two-hybrid screen identified several other SRPX2 interactors: a GPI-anchored plasminogen activator receptor named uPAR, the cysteine protease cathepsin B and the metalloproteinase ADAMTS4 (Royer-Zemmour et al., 2008). Notably, all these proteins are components of the extracellular proteolysis machinery, suggesting that, in addition to its regulation of the complement, the role of SRPX2 in synaptogenesis could involve remodeling of the extracellular matrix, thereby regulating the clustering of glutamate receptors.

#### SUSD2 and SUSD4

The Sushi domain-containing protein (SUSD) family is composed of six proteins, which all contain one or more CCP domains (Figure 2), and, except SUSD1, a transmembrane domain. The *Susd2* and *Susd3* genes also encode a shorter secreted isoform. SUSD2 was the first member with a described function at neuronal synapses (Nadjar et al., 2015) and localizes in somata and dendrites of cultured hippocampal neurons. Knockdown of *Susd2* results in increased dendritic length, reduced axon length and branching and reduced excitatory synapse numbers (Nadjar et al., 2015). Loss-of-function of *Susd4* in mice leads to motor coordination adaptation and learning impairments (Zhu et al., 2020; González-Calvo et al., 2021) and misregulation of synaptic plasticity in cerebellar Purkinje cells with decreased long-term depression (González-Calvo et al., 2021). SUSD4 interacts *via* its cytoplasmic domain with several HECT ubiquitin ligases of the NEDD4 subfamily (González-Calvo et al., 2021). These ubiquitin ligases promote the ubiquitination and degradation of a large number of cellular substrates, including AMPA Receptors (Schwarz et al., 2010; Zhu et al., 2017). Loss-of-function of *Susd4* prevents activity-dependent degradation of GluA2 AMPA receptor subunits after chemical LTD induction (González-Calvo et al., 2021). Thus interaction of SUSD4 with GluA2 and NEDD4 ubiquitin ligases could promote the targeting of AMPA receptors to the degradation compartment and long-term synaptic depression (González-Calvo et al., 2021). Interestingly, the extracellular domain of SUSD4, containing the CCP domains, interacts strongly with the GluA2-containing AMPA receptors (González-Calvo et al., 2021) : given the existence of potential cleavage sites that could release the extracellular domain, further work should aim at testing whether this domain alone regulates the localization and/or function of GluA2 subunits. Besides *SUSD4, SUSD1*, *SUSD5* and *SUSD6* are expressed in the human primary motor cortex with the highest expression level found in excitatory neurons (Annex 1), suggesting a role for these other SUSD members in brain development and/or function.

#### Proteins of the Extracellular Matrix

The extracellular matrix plays an active role in synapse function and plasticity (reviewed in (Frischknecht et al., 2014)) and contains several CCP domain-containing proteins: Aggrecan (ACAN), Brevican (BCAN), Neurocan (NCAN, also known as CSPG3) and Versican (VCAN). All four proteins contain a single CCP domain at their C-terminus (Figure 2) and are found in perineuronal nets, a specialized structure of the extracellular matrix around various types of neurons (Fawcett et al., 2019). In the absence of BCAN, perineuronal nets around neurons of the hippocampus are disorganized and sparse (Brakebusch et al., 2002). Synapses between the Schaffer collaterals and the CA1 pyramidal neurons in *Bcan* knockout mice are dramatically impaired for the maintenance of synaptic long-term potentiation (LTP), while the basic properties of excitatory and inhibitory synapses as well as LTP induction are normal (Brakebusch et al., 2002). In the *Ncan* knockout mice, maintenance of synaptic LTP in the CA1 region is impaired while the perineuronal nets appear largely normal (Zhou et al., 2001). Experiments in cultured neurons indicate that these proteins might also contribute to the regulation of synapse numbers (Geissler et al., 2013; Gottschling et al., 2019). CCP domain-containing proteins in the extracellular matrix are thus essential for the formation of perineuronal nets and the formation and function of synapses, but the role of the CCP domain in these proteins remains to be deciphered.

### CUB-Domain Containing Proteins: From Auxiliary Subunits to Synapse Formation

#### NETO1 and NETO2

NETO1 and NETO2 are brain-specific proteins with a domain organization resembling the one of CUB-domain containing proteins in invertebrates (LEV-10, SOL-2 and *Drosophila* Neto). They are both type 1 transmembrane proteins with a large ectodomain composed of one LDLa domain and two CUB domains (Stohr et al., 2002; Michishita et al., 2003, 2004). The C-terminal region of NETO1 and NETO2 contains a class I- and class II- PDZ binding motif, respectively (Ng et al., 2009; Tang et al., 2012; Figure 2). The CUB domains of NETO proteins are required for their interaction with NMDARs (Ng et al., 2009), kainate receptors KARs (Tang et al., 2011) and the K^+^-Cl^–^ cotransporter KCC2 (Ivakine et al., 2013). NETO2 interacts with GRIP through its C-terminal PDZ binding motif (Tang et al., 2012) while NETO1 interacts with the scaffolding protein PSD-95 (Ng et al., 2009). Both NETO1 and NETO2 are enriched in biochemical fractions enriched in postsynaptic densities (Ng et al., 2009; Zhang et al., 2009).

In vertebrates, NETO1 and NETO2 have been primarily studied for their role as auxiliary subunits of kainate receptors (Zhang et al., 2009; Copits et al., 2011; Straub, 2011; Fisher and Mott, 2013). Coexpression of Neto1 or Neto2 with kainate receptors in heterologous cells greatly enhances glutamate-evoked currents (Zhang et al., 2009). More specifically, Neto1 increases KAR affinity for kainate and glutamate (Straub, 2011; Fisher and Mott, 2013), increases KAR EPSC amplitude (Straub, 2011; Tang et al., 2011), and regulates KAR desensitization properties (Copits et al., 2011; Straub, 2011; Fisher and Mott, 2013; Sheng et al., 2015). However, exactly how NETO proteins regulate KARs may vary depending on the subunit composition and on the synapse. NETO1 and NETO2 have a different pattern of expression in the brain. NETO2 is very abundant in the cerebellum, while NETO1 is highly expressed in the hippocampus, particularly in the CA3 field (Michishita et al., 2003, 2004; Ng et al., 2009; Straub, 2011; Tang et al., 2011, 2012). Indeed, KAR mediated currents are reduced at the mossy fiber/CA3 synapse of *Neto1*^–/–^ mice but not *Neto2*^–/–^ mice (Tang et al., 2011). Moreover, NETO1, but not NETO2, regulates KAR currents in hippocampal interneurons (Wyeth et al., 2017). In addition to the defects in kainate mediated currents, *Neto1*^–/–^ mice present NMDAR defects at specific synapses: NMDAR mediated currents are reduced at CA1/Schaffer collateral synapses and at associational/commissural CA3 synapses but not at mossy fiber CA3 synapses (Ng et al., 2009; Straub, 2011; Tang et al., 2011). No defects in AMPAR mediated currents have been detected in the absence of *Neto1* or *Neto2* (Ng et al., 2009; Zhang et al., 2009).

Besides their role in regulating glutamate receptor function, NETO proteins could also regulate receptor localization, although the evidence for this role is contradictory. While some studies did not detect any changes in KAR abundance at synapses in the absence of NETO proteins (Zhang et al., 2009; Straub, 2011), others suggest that NETO proteins may be involved in the recruitment and/or stabilization of KARs at synapses (Tang et al., 2011, 2012; Wyeth et al., 2014; Mennesson et al., 2019). In *Neto1* knockout mice, GluN2A levels are reduced in PSD fractions but not in whole brain extract, and surface GluN2A are unchanged (Ng et al., 2009). This suggests that NETO1 could have a role in clustering of GluN2A at glutamatergic synapses, reminiscent of the role of CUB-domain containing proteins in invertebrates. Furthermore, the absence of *Neto1* leads to loss of presynaptic kainate receptors at immature CA3-CA1 synapses and a deficit in synaptogenesis (Orav et al., 2017). Thus, besides their role as auxiliary subunits of glutamate receptors, NETO proteins could also contribute to the regulation of synapse formation and molecular composition.

#### Neuropilin-1 and 2

The Neuropilin family is composed of two members, neuropilin-1 and neuropilin-2 (NRP-1 and NRP-2, respectively, Figure 2). Both neuropilins are transmembrane glycoproteins with one short cytosolic domain, a MAM domain (for meprin, A-5 protein and receptor protein tyrosine phosphatase mu), a discoidin domain and two N-terminal CUB domains (Kolodkin et al., 1997). Neuropilins are the receptors for class 3 secreted semaphorins: NRP-1 binds with high affinity to semaphorin 3A (SEMA3A) and NRP-2 binds to SEMA3F. While they were first described as receptors for axon guidance cues (Kolodkin et al., 1997), both are enriched in synaptosomal and PSD preparations from the adult hippocampus and have been shown to play synaptic roles. In the cerebellum, Basket cells express NRP-1 and Purkinje cells express its ligand SEMA3A (Telley et al., 2016). Proper contact and synapse development between Purkinje cells and basket cell axons require the interaction of NRP-1 with the adhesion protein NF186. This interaction is facilitated by SEMA3A secretion in Purkinje cells (Telley et al., 2016). While the CUB domain of NRP-1 is involved in the interaction with its ligand SEMA3A, whether it is involved in additional aspect of NRP-1 function at synapses remain to be determined. Knockout for *Nrp2* in mice leads to increased epileptogenicity (Eisenberg et al., 2021). Increased spine densities and changes in spine morphogenesis are found in both mouse knockout for *Nrp-2* and *Sema3F*, a ligand of NRP2 (Tran et al., 2009). In the *Nrp-2* knockout mice, patch-clamp recordings found increased mEPSC frequency in cortical and hippocampal neurons in the absence of changes in the paired-pulse ratio (Tran et al., 2009) and decreased mIPSCs in CA1 neurons accompanying a reduction in interneuron numbers (Eisenberg et al., 2021), indicating an effect on the number of synapses. In addition, loss-of-function of NRP-2 prevents homeostatic plasticity since it prevents bicuculline-induced reduction in surface AMPA receptors and in synaptic strength in cortical neurons. NRP-2 is found in spines of cultured cortical neurons where it colocalizes with the GluA1 subunit of AMPA receptors (Wang et al., 2017). NRP-2 interacts with GluA1-containing AMPA receptors through its CUB domains, and this interaction is modulated by neuronal activity and SEMA3F (Wang et al., 2017). These results show that the CUB domains of NRP-2 are key for homeostatic plasticity at excitatory synapses by enabling interaction with GluA1 AMPA receptor subunits.

## Addition and Combination of Structural Domains in Vertebrate Synaptic Proteins Accompanies the Complexification of Synapses

**FIGURE 3:**
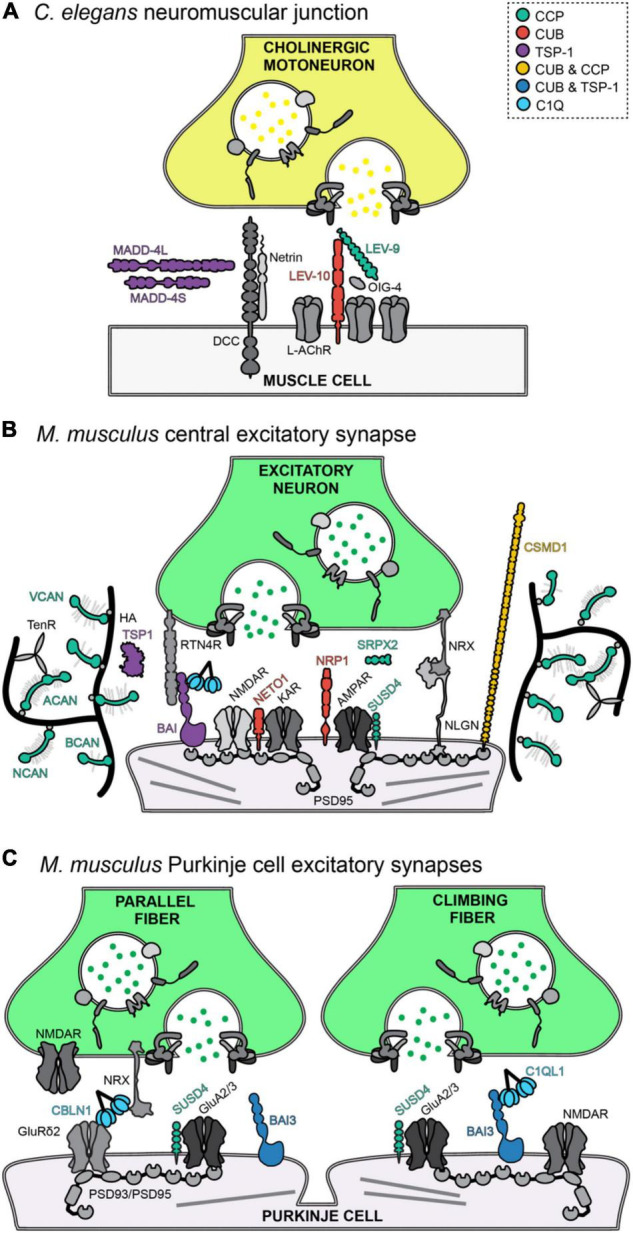
CCP-, CUB-, and TSP-1-domain containing proteins in *C. elegans* and in mammalian synapses. **(A)** In *C. elegans*, the three classes of proteins are found in cholinergic neuromuscular junctions and play different functions in synapse specification (MADD-4) and receptor clustering (LEV-9 and LEV-10). **(B)** In *M. musculus*, the three domains are found either alone (SUSD4, NETO1, …) or in combination (CSMD1, BAI3) in proteins of excitatory synapses. **(C)** The C1Q domain, absent in *C. elegans*, is found in proteins that specify two different types of excitatory synapses made on a single target, the Purkinje cell.

Comparing the structure of CCP, CUB, and TSP-1-containing proteins across species shows that invertebrates contain synaptic proteins with stretches of a single domain type whereas mammalian synaptic proteins are composed of combinations of CCP, CUB, and TSP-1 domains, together with a large diversity of other domains (Figure 2). This suggests that multiple functions have been combined in vertebrates in single proteins and that the function of a particular domain could be partially inferred by the function of invertebrate proteins. CCP containing proteins in invertebrates such as LEV9 (Figure 3), Hig and Hasp play a major role in the clustering of synaptic proteins, in particular neurotransmitter receptors. CUB domain containing proteins in invertebrates, such as SOL1 (Figure 3) or dSOL1, function as auxiliary subunit and/or control receptor gating. The phenotype of mouse mutants for CCP or CUB domain-containing proteins indicate that their function might be indeed conserved: CCP proteins such as SRPX2 and SUSD4 play roles in controlling receptor numbers at synapses while CUB containing NETO proteins control kainate receptor gating. The studies of MADD-4 in *C. elegans* suggest a role for TSP-1 containing proteins in synapse specification on a given target cell (Figure 3) and the Thrombospondins and BAI proteins in mammals play a role in synaptogenesis and receptor localization. Conversely, studies in mammals can reveal the importance of structural domains that are not found in invertebrates. For example the C1q globular domain defines a large family of proteins that includes proteins with known synaptic functions: the innate immunity protein C1Q that also regulates synapse elimination in the nervous system, the cerebellins and C1QL proteins (Bolliger et al., 2011; Yuzaki, 2011; Sigoillot et al., 2015). C1QL1 is specifically secreted by one type of excitatory input of the cerebellar Purkinje cells, the climbing fibers, and is essential, together with its receptor BAI3, for excitatory synapse specification in cerebellar Purkinje cells (Figure 3). The C1q globular domain is not found in *C. elegans*, suggesting that the appearance of this structural domain might have contributed to the increased diversity of synapse types in vertebrates. Interestingly, while CCP, CUB, and C1Q domain containing proteins were identified first in the immune system, the presence of CCP and CUB domain proteins in *C. elegans* suggest an ancient role for these structural domains in forming and regulating signaling complexes, in particular with neurotransmitter receptors, a role that could have been then tethered to various biological systems during evolution.

CCP and CUB domains are combined in neuronal proteins such as SEZ6 isoforms (Shimizu-Nishikawa et al., 1995; Osaki et al., 2011) and CSMD1-3 (CUB and Sushi multiple domains family of proteins; Figure 2). Neurons lacking SEZ-6 present fewer spines, PSD95 puncta and reduced EPSPs (Gunnersen et al., 2007), suggesting a yet to be understood synaptic function. All three CSMD proteins have fourteen CUB domains and twenty-six to twenty-eight CCP domains (Figure 2). CSMD1 and CSMD2 are both synaptic (Loh et al., 2016; Gutierrez et al., 2019). CSMD2 directly interacts with the scaffold PSD-95 through its PDZ-binding domain and knockdown of CSMD2 reduces spine density in cultured hippocampal neurons (Gutierrez et al., 2019). Thus, both for SEZ-6 and CSMD proteins, their precise synaptic function, and in particular, whether the functions of CUB and CCP domains in clustering and gating receptors are conserved, remains to be demonstrated. Interestingly, TSP-1 domains in synaptic proteins are not associated to CUB or CCP domains, except for BAI3 that contains a single CUB domain at its N-terminus (Figure 2). Thus, synapse specification on one hand and receptor clustering and gating on the other hand might be two synaptic aspects controlled independently by proteins containing TSP-1 and CUB/CCP domains, respectively. To demonstrate this hypothetical division of labor, further studies of the precise roles of independent domains in mammalian synaptic proteins using mutagenesis are warranted.

The percentage of proteins containing CCP, CUB, and TSP-1 domains is increased in vertebrates compared to invertebrates (Figure 1). Many families of such proteins are highly expressed in the human brain (Annex 1), but their role remains to be determined. In addition, while the role of proteins containing CCP, CUB, and TSP-1 domains at excitatory synapses in mammals starts to be better understood, their role at inhibitory synapses remains largely unknown and deserves further study. In many cases, mutations in genes coding CCP, CUB, or TSP-1 containing proteins in humans have been associated with brain diseases highlighting their importance for brain development and function. In *Homo sapiens*, the *SUSD4* gene is located in the chromosome deletion linked with Fryns syndrome, which is an autosomal recessive multiple congenital neurodevelopmental disorder associated with intellectual disability (Shaffer et al., 2007) and *SUSD4* variants have also been associated with autism spectrum disorders (Cuscó et al., 2009; Coe et al., 2019). *ADGRB1-3* genes have been associated with disorders such as autism spectrum disorders (Michaelson et al., 2012), schizophrenia (DeRosse et al., 2008), bipolar disorder (McCarthy et al., 2012), intellectual disability (Scuderi et al., 2019) and addiction (Liu et al., 2006). Variants in *ADAMTSL3*, the human orthologue of *C. elegans* MADD-4, and all three *CSMD* genes have been associated with schizophrenia (Need et al., 2009; Dow et al., 2011; Schizophrenia Psychiatric Genome-Wide Association Study (GWAS) Consortium, 2011; Luo et al., 2018). Understanding the specific functions of the CCP, CUB, or TSP-1 containing proteins is thus an emerging field with direct relevance for the treatment of brain diseases.

## Author Contributions

IG-C, MC, J-LB, and FS contributed to conception of the manuscript. IG-C, MC, and FS wrote sections of the manuscript. All authors contributed to manuscript revision, read, and approved the submitted version.

## Conflict of Interest

The authors declare that the research was conducted in the absence of any commercial or financial relationships that could be construed as a potential conflict of interest.

## Publisher’s Note

All claims expressed in this article are solely those of the authors and do not necessarily represent those of their affiliated organizations, or those of the publisher, the editors and the reviewers. Any product that may be evaluated in this article, or claim that may be made by its manufacturer, is not guaranteed or endorsed by the publisher.

## Footnotes

1
smart.embl.de
