# Supplementary figures and images for: Synapse Formation and Function Across Species: Ancient Roles for CCP, CUB, and TSP-1 Structural Domains
Source: Front Neurosci. 2022 Apr 25;16:866444. doi: 10.3389/fnins.2022.866444 (PMC9083331; doi:10.3389/fnins.2022.866444)

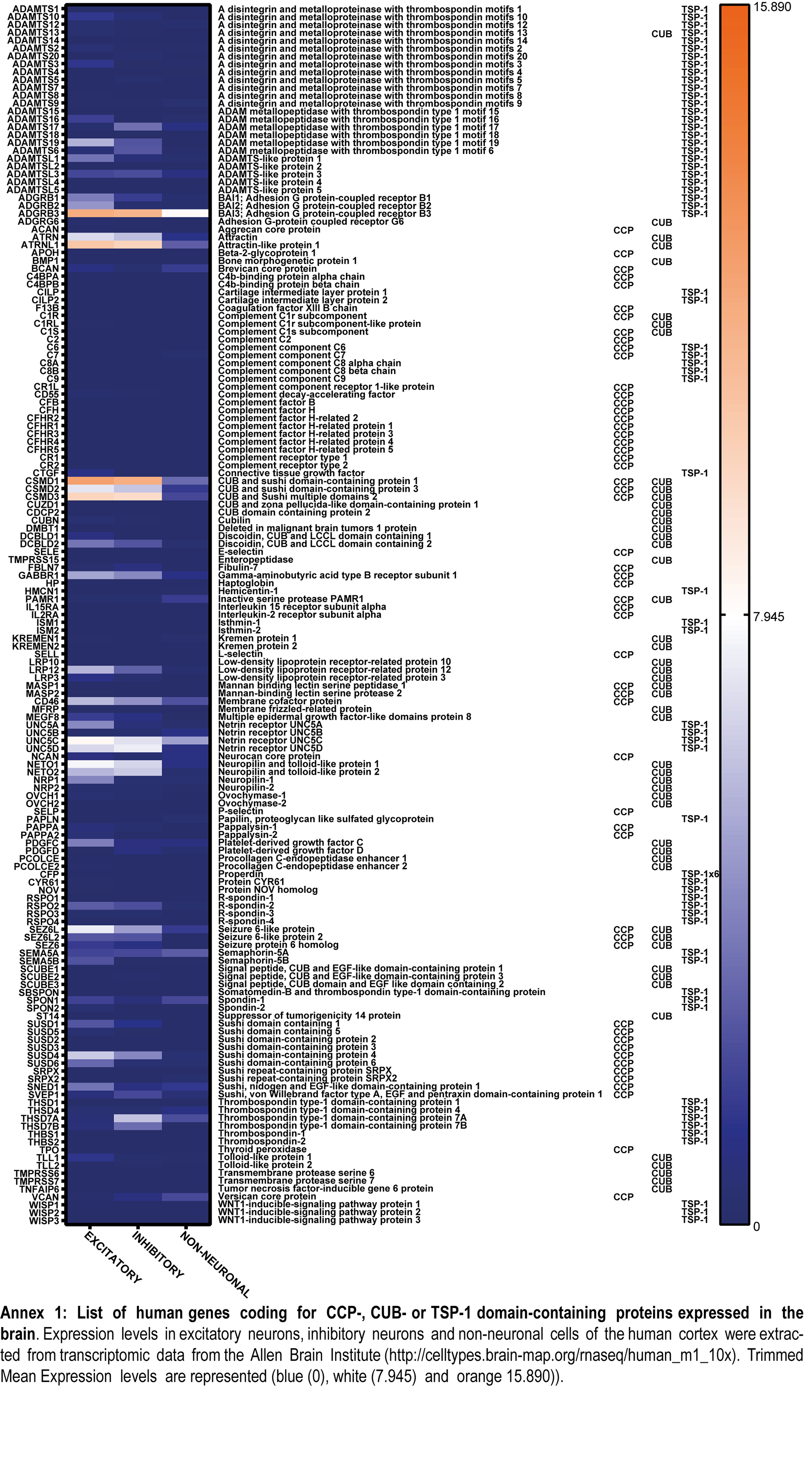

Supplement: Supplementary file 1 [file Image_1.tif]
